# Personal

**Published:** 1902-11

**Authors:** 


### PERSONAL.

HON. JAMES O. PUTNAM, who has been a member of the council of the University of Buffalo since its foundation, and for many years past the chancellor, recently resigned the latter office because of the infirmities of advancing age. The council accepted Mr. Putnam's resignation and voted that the following comment upon it be placed on the records:

The long and devoted services which the Honorable James O. Putnam has rendered to the University of Buffalo deserve special and grateful recognition at our hands, particularly at a time when the infirmities incident to advancing years have made it seem wise that he be relieved of the burdens of the responsible office of chancellor of said university.

It is with very keen regret that we accept Mr. Putnam's resignation and trust that he may still be spared for many years of usefulness in the council.

DR. M. D. MANN, of Buffalo, who attended the recent international gynecologic congress at Rome, has returned and resumed his professional work.

DR. DANIEL LEWIS, of New York, Dr. CHARLES A. L. REED, of Cincinnati, and Dr. JOHN E. RHODES, of Chicago, the committee appointed by the Maltine Company to award the prizes offered by that house for the best essays on preventive medicine, recently held their final meeting in Buffalo. It is probable their findings soon will be announced.

DR. REGINA FLOOD KEYES recently was appointed a member of the medical staff of the Erie County Hospital, vice Dr. Earl P. Lothrop, resigned. Dr. Keyes is also assistant gynecologist at the Buffalo General Hospital and clinical instructor at the University of Buffalo.

---

Dr. Mary Clayton, of Buffalo, has been appointed to a place in the medical service of the St. Lawrence State Hospital, and has entered upon her duties there.

---

Dr. Marian Marsh, formerly of Buffalo, late of Jamestown, has returned to this city and opened offices at 2148 Main Street for the practice of her profession.

---

Dr. Adolph Lorenz, professor of orthopedic surgery in the University of Vienna, is visiting this country and has recently held orthopedic clinics at several hospitals in Chicago. The special object of his visit was to operate upon a little daughter of J. Ogden Armour, for which it is reported he received a handsome fee. At the clinics referred to, however, he rendered gratuitous services.

---

Dr. Herman Mynter, of Buffalo, who with his family made an extended tour in Europe during the past summer, has returned and taken up his accustomed surgical work.

